# Optimizing patient outcomes in severe pneumonia: the role of multiplex PCR in the treatment of critically ill patients

**DOI:** 10.3389/fmed.2024.1391641

**Published:** 2024-08-21

**Authors:** Jia-Hao Zhang, San-Fang Chou, Ping-Huai Wang, Chia-Jui Yang, Yi-Horng Lai, Mei-Yun Chang, Hou-Tai Chang

**Affiliations:** ^1^Department of Critical Care Medicine, Far Eastern Memorial Hospital, Taipei, Taiwan; ^2^Department of Nursing, Cardinal Tien College of Healthcare and Management, New Taipei, Taiwan; ^3^Department of Chest Medicine, Far Eastern Memorial Hospital, Taipei, Taiwan; ^4^Department of Medical Research, Far Eastern Memorial Hospital, Taipei, Taiwan; ^5^Department of Infection Medicine, Far Eastern Memorial Hospital, Taipei, Taiwan; ^6^Department of Healthcare Administration, Asia Eastern University of Science and Technology, Taipei, Taiwan; ^7^Department of Industrial Engineering and Management, Yuan Ze University, Taoyuan, Taiwan

**Keywords:** BioFire^®^ FilmArray, intensive care unit, pneumonia, respiratory tract infections, APACHE-II score, SOFA score

## Abstract

Herein, we evaluated the optimal timing for implementing the BioFire^®^ FilmArray^®^ Pneumonia Panel (FA-PP) in the medical intensive care unit (MICU). Respiratory samples from 135 MICU-admitted patients with acute respiratory failure and severe pneumonia were examined using FA-PP. The cohort had an average age of 67.1 years, and 69.6% were male. Notably, 38.5% were smokers, and the mean acute physiology and chronic health evaluation-II (APACHE-II) score at initial MICU admission was 30.62, and the mean sequential organ failure assessment score (SOFA) was 11.23, indicating sever illness. Furthermore, 28.9, 52.6, and 43% of patients had a history of malignancy, hypertension, and diabetes mellitus, respectively. Community-acquired pneumonia accounted for 42.2% of cases, whereas hospital-acquired pneumonia accounted for 37%. The average time interval between pneumonia diagnosis and FA-PP implementation was 1.9 days, and the mean MICU length of stay was 19.42 days. The mortality rate was 50.4%. Multivariate logistic regression analysis identified two variables as significant independent predictors of mortality: APACHE-II score (*p* = 0.033, OR = 1.06, 95% CI 1.00–1.11), history of malignancy (OR = 3.89, 95% CI 1.64–9.26). The Kaplan–Meier survival analysis indicated that early FA-PP testing did not provide a survival benefit. The study suggested that the FA-PP test did not significantly impact the mortality rate of patients with severe pneumonia with acute respiratory failure. However, a history of cancer and a higher APACHE-II score remain important independent risk factors for mortality.

## 1 Introduction

According to statistics from the World Health Organization (WHO), lower respiratory tract infections and pneumonia are the fourth leading cause of death worldwide. Moreover, pneumonia is a prevalent condition encountered in intensive care units. Pneumonia-related mortality is higher in elderly patients and individuals with a history of malignancy (1, 2). In clinical practice guidelines provided by various organizations, the recommended antibiotic choices for patients with severe pneumonia include penicillin, fluoroquinolones, or agents effective against methicillin-resistant *Staphylococcus aureus* (MRSA). The extensive use of antibiotics, however, has contributed to the emergence of drug-resistant bacteria, causing a shift in the spectrum of pathogenic bacteria involved in community- or hospital-acquired pneumonia. Further, the prevalence of pneumonia caused by multidrug-resistant (MDR) bacteria ranges from 14.1 to 62%, leading to increased morbidity and mortality among patients, and imposing a substantial economic burden in terms of social costs (3–5). According to the American Thoracic Society/Infectious Disease Society of America (ATS/IDSA), sputum Gram staining, aerobic culture, and blood culture collection should be performed prior to administering broad-spectrum antibiotics. This approach aims to ensure an accurate diagnosis and appropriate treatment. In addition, compliance with these guidelines can help to prevent the overuse of broad-spectrum antibiotics, a critical factor in the development of antibiotic resistance (6). However, it is important to note that only 31.9% of the patients diagnosed with community-acquired pneumonia (532 of 1,669) managed to produce high-quality sputum samples. Additionally, among these cases, only 14.4% (240 of 1,669) of the collected sputum samples could be cultured to identify the predominant morphotype. The limited availability of high-quality sputum samples poses challenges in accurately diagnosing and treating pneumonia (7). Moreover, traditional sputum cultures generally require a waiting period of 2–5 days before the final report is available. In contrast, the utilization of a rapid diagnostic test, such as the BioFire^®^ FilmArray^®^ Pneumonia Panel (FA-PP; BioFire Diagnostics, Salt Lake City, UT, United States), which employs multiplex polymerase chain reaction (PCR) technology, offers a significantly higher detection rate (ranging from 74.6 to 92%) than sputum cultures obtained from endotracheal aspirate or bronchoalveolar lavage samples. This expeditious and more accurate diagnostic approach could facilitate prompt and targeted treatment decisions for patients with pneumonia (8–10).

The FA-PP can detect eight viruses, 18 bacteria, and seven antibiotic resistance genes within a remarkably short timeframe of only 60 min, offering a rapid and comprehensive diagnostic solution for patients with pneumonia. Hence, the implementation of a multiplex PCR system to analyze respiratory samples from patients with hospital-acquired and ventilator-associated pneumonia has the potential to improve empirical antimicrobial therapy and reduce the use of broad-spectrum antibiotics. This change could increase the de-escalation rate from 39 to 48.2% (8, 11, 12).

However, the impact of multiplex PCR on the mortality rate of patients with severe pneumonia in the intensive care unit remains uncertain. In addition, the optimal timing of application in cases of severe pneumonia remains unknown. Hence, this retrospective cohort study sought to ascertain the most effective timing for implementing multiplex polymerase chain reaction (PCR) in the management of patients with severe pneumonia, and to assess its potential to improve the survival rate of these patients.

## 2 Materials and methods

This retrospective single-center cohort study was conducted at a medical center in Taiwan between July 1, 2021 and July26, 2022. This study used anonymous data and was approved by the Medical Ethics Committee of the Far Eastern Memorial Hospital (approval number: 111211-E).

Patients admitted to the medical intensive care unit (MICU) with acute respiratory failure and severe pneumonia were included in accordance with the diagnostic criteria outlined by the Infectious Diseases Society of America (IDSA). These criteria necessitated the presence of either new or progressive chest X-ray consolidations combined with clinical symptoms, such as dyspnea, cough, sputum production, fever, and abnormal breathing sounds indicative of pulmonary consolidation. All the above conditions were important criteria for enrolling study patients (6, 13). Specific exclusion criteria were implemented to maintain a focused analysis. Patients who did not receive invasive mechanical ventilation were excluded.

Respiratory samples were collected via tracheal aspiration (TA) or bronchoalveolar lavage (BAL). Two distinct methods were employed for the sample analysis. The first involved traditional microbiological techniques, including sputum Gram staining and aerobic culturing. The second employed a multiplex PCR system, FA-PP, operated according to the manufacturer’s recommended protocol.

Clinical data were collected retrospectively and anonymized. The collected data encompassed various patient aspects, including age, gender, pneumonia type (e.g., community-acquired pneumonia (CAP), healthcare-associated pneumonia (HCAP), hospital-acquired pneumonia (HAP), and ventilator-associated pneumonia (VAP)), acute physiology and chronic health evaluation-II (APACHE-II) score, sequential organ failure assessment (SOFA) scores, serum lactate level, smoking history, major underlying medical conditions (e.g., malignancy, diabetes mellitus, hypertension, etc.), time interval between pneumonia diagnosis and FA-PP procedure, intubation duration, length of stay, and in-hospital mortality. The term HCAP indicates that patients must meet the diagnosis of pneumonia and also have one of the following conditions: hospitalization in an acute care hospital for more than 2 days within the past 90 days, residing in a nursing home or long-term care facility, receiving intravenous antibiotics, chemotherapy, or dialysis within the past 30 days. HAP refers to patients meeting the diagnosis of pneumonia occurring more than 48 h after hospital admission, or within 14 days after discharge from a previous hospitalization. Factors that influenced mortality and survival outcomes in patients with severe pneumonia who underwent FA-PP treatment, were subsequently identified.

### 2.1 Statistical analysis

Categorical data were expressed as frequency and percentage, while numerical data were expressed as mean ± standard deviation (SD) or median (1st quartile, 3rd quartile) (Q1, Q3). The difference between groups was examined using independent-samples *T* test or Mann–Whitney U test for continuous data. The Pearson Chi-Square test was used to compare differences between groups for categorical data, unless otherwise noted. We further performed multivariate logistic regression analysis to identify potential risk factors associated with mortality in patients with severe pneumonia. Kaplan–Meier analysis was performed to assess the 28-day survival and in-hospital survival. The Kaplan–Meier survival curves were compared using the log-rank test. Statistical analysis was conducted using IBM SPSS Statistics 27 software. The results were considered statistically significant at the *p* < 0.05 level.

## 3 Results

**Figure 1:**
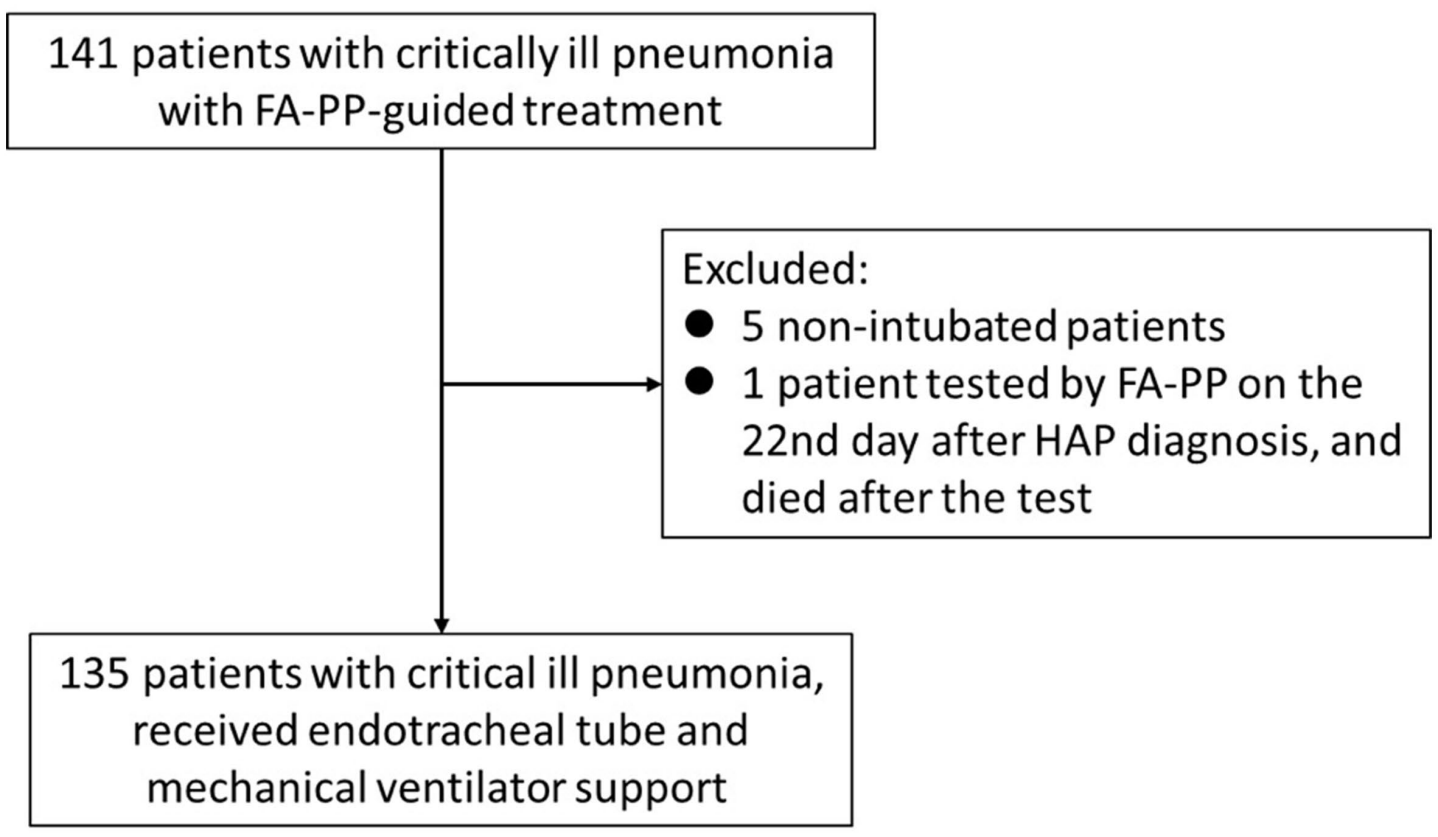
Flow chart of the screening process.

Data were initially collected from 141 patients between July 2021 and July 2022. The exclusion criteria were applied as follows: five patients were excluded for not receiving tracheal intubation therapy; one outlier was excluded because the FA-PP test was performed 22 days after being diagnosed with HAP, and the patient died after the FA-PP test was conducted. Finally, 135 patients were enrolled in this study (Figure 1). An overview of the basic characteristics and clinical outcomes of the 135 patients who underwent FA-PP testing for pathogens and received mechanical ventilation support at the time of enrollment was shown in Table 1. The patient cohort had an average age of 67.10 ± 13.92 years, with 69.6% male. Of the 135 patients, 38.5% had a prior history of smoking, and the averages of severity indexes, including APACHE-II score, SOFA score, and serum lactate, were 30.62 ± 8.46, 11.23 ± 3.88, and 4.50 ± 4.19, respectively. Community-acquired pneumonia accounted for 42.2% of pneumonia cases, whereas HAP accounted for 37.0%. The time interval between pneumonia diagnosis and FA-PP implementation was 1.90 ± 1.62 days, and the average length of MICU stay was 19.42 ± 12.93 days. The overall in-hospital mortality rate was 50.4%.

**Table 1:** Baseline demographic characteristics and clinical outcomes of critically ill patients.

Patient characteristics	Results	Min–Max
Age, years, mean±SD	67.10±13.92	22–97
Sex		
Male, *n* (%)	94 (69.6)	
Female, *n* (%)	41 (30.4)	
Smoker, *n* (%)	52 (38.5)	
APACHE-II score, mean±SD	30.62±8.46	14–57
SOFA score, mean±SD	11.23±3.88	3–21
Serum lactate, mmol/L, mean±SD	4.50±4.19	0–22.33
Comorbidities		
Malignancy, *n* (%)	39 (28.9)	
Chronic obstructive pulmonary disease, *n* (%)	15 (11.1)	
Asthma, *n* (%)	1 (0.7)	
Bronchiectasis, *n* (%)	2 (1.5)	
Hypertension, *n* (%)	71 (52.6)	
Diabetes mellitus, *n* (%)	58 (43.0)	
Coronary artery disease, *n* (%)	27 (20.0)	
Congestive heart failure, *n* (%)	22 (16.3)	
End stage renal disease, *n* (%)	15 (11.1)	
Liver cirrhosis, *n* (%)	12 (8.9)	
Type of pneumonia		
Community-acquired pneumonia, *n* (%)	57 (42.2)	
Healthcare-associated pneumonia, *n* (%)	15 (11.1)	
Hospital-acquired pneumonia, *n* (%)	50 (37.0)	
Ventilator-associated pneumonia, *n* (%)	13 (9.6)	
Interval time between pneumonia diagnosis and FA-PP, days, mean±SD/median (Q1, Q3)	1.90±1.62/1 (1, 2)	1–10
Outcome parameter		
Duration of intubation, mean±SD	21.56±18.98	1–101
ICU length of stay, mean±SD	19.42±12.93	2–65
Hospital length of stay, mean±SD	37.08±27.15	2–159
In-hospital mortality, *n* (%)	68 (50.4)	

Numerical data was expressed as mean ± SD or median (Q1, Q3) while categorical data was expressed as frequency and percentage. APACHE-II, acute physiology and chronic health evaluation-II; SOFA, sequential organ failure assessment; FA-PP, BioFire^®^ FilmArray^®^ pneumonia panel; ICU, intensive care unit.

The differences in basic characteristics and clinical outcomes between the survived and deceased groups were examined. The results suggested that APACHE-II score, SOFA score, history of malignancy, and the time interval between pneumonia diagnosis and FA-PP might have been the risk factors associated with the mortality (Table 2). To reveal the impact of those factors on the mortality, multivariate logistic analysis was performed (Table 3). APACHE-II score and history of malignancy were associated with death, with ORs of 1.06 (*p* = 0.033) and 3.89 (*p* = 0.002), respectively. However, the time interval between pneumonia diagnosis and the FA-PP test did not reach statistical significance (OR = 1.24, *p* = 0.067).

**Table 2:** Association between risk factors and mortality among critically ill patients with pneumonia who underwent FA-PP-guided treatment.

Variable	Survival (*n* = 67)	Death (*n* = 68)	*p*-value
Age, years, mean±SD	65.67±16.21	68.50±11.15	0.241
Sex			0.896
Male, *n* (%)	47 (70.1)	47 (69.1)	
Female, *n* (%)	20 (29.9)	21 (30.9)	
Smoker, *n* (%)	30 (44.8)	22 (32.4)	0.138
APACHE-II score, mean±SD	28.79±7.94	32.43±8.63	0.012*
SOFA score, mean±SD	10.19±3.78	12.25±3.73	0.002*
Serum lactate, mmol/L, mean±SD	4.03±3.81	4.96±4.51	0.196
Comorbidities			
Malignancy, *n* (%)	11 (16.4)	28 (41.2)	0.002*
Chronic obstructive pulmonary disease, *n* (%)	8 (11.9)	7 (10.3)	0.761
Asthma, *n* (%)	0 (0.0)	1 (1.5)	1.000a
Bronchiectasis, *n* (%)	1 (1.5)	1 (1.5)	1.000a
Hypertension, *n* (%)	34 (50.7)	37 (54.4)	0.670
Diabetes mellitus, *n* (%)	34 (50.7)	24 (35.3)	0.070
Coronary artery disease, *n* (%)	13 (19.4)	14 (20.6)	0.836
Congestive heart failure, *n* (%)	13 (19.4)	9 (13.2)	0.332
End stage renal disease, *n* (%)	9 (13.4)	6 (8.8)	0.394
Liver cirrhosis, *n* (%)	5 (7.5)	7 (10.3)	0.764a
Type of pneumonia			0.038*b
Community-acquired pneumonia, *n* (%)	32 (47.8)	25 (36.8)	
Healthcare-associated pneumonia, *n* (%)	9 (13.4)	6 (8.8)	
Hospital-acquired pneumonia, *n* (%)	24 (35.8)	26 (38.2)	
Ventilator-associated pneumonia, *n* (%)	2 (3.0)	11 (16.2)	
Interval time between pneumonia diagnosis and FA-PP, days, mean±SD/median (Q1, Q3)	1.67±1.40/1 (1, 2)	2.13±1.79/1.5 (1, 2)	0.098/0.035*c
Outcome parameter			
Duration of intubation, mean±SD	19.15±14.92	23.94±22.13	0.142
ICU length of stay, mean±SD	17.76±10.25	21.06±15.01	0.138
Hospital length of stay, mean±SD	37.73±20.61	36.44±32.49	0.783

Numerical data was expressed as mean ± SD or median (Q1, Q3) while categorical data was expressed as frequency and percentage. The differences between 2 groups were analyzed using either Independent-Samples *T* test, Mann–Whitney U test^c^ or Chi-Square test, depending on the data was numerical or categorical. a, Fisher’s Exact test used due to more than one cell count < 5 or < 20% in 2×2 crosstab. b, Chi-Square/Likelihood Ratio test used due to more than one cell count < 5 or < 20% in crosstab more than 2×2 cells. APACHE-II, acute physiology and chronic health evaluation-II; SOFA, sequential organ failure assessment; FA-PP, BioFire^®^ FilmArray^®^ pneumonia panel; ICU, intensive care unit. *, *p* value less than 0.05 indicates statistical significance.

**Table 3:** Multivariate logistic regression analysis of the association between risk factors and mortality among critically ill patients with pneumonia receiving FA-PP-guided treatment.

Variable	Multivariate logistic regression	
	OR (95% CI)	*p*-value
APACHE-II score	1.06 (1.00–1.11)	0.033*
SOFA score	1.11 (0.99–1.24)	0.067
Malignancy	3.89 (1.64–9.26)	0.002*
Interval time between pneumonia diagnosis and FA-PP, day	1.24 (0.99–1.56)	0.067

APACHE-II, acute physiology and chronic health evaluation-II; SOFA, sequential organ failure assessment; FA-PP, BioFire^®^ FilmArray^®^ pneumonia panel. *, *p* value less than 0.05 indicates statistical significance.

**Figure 2:**
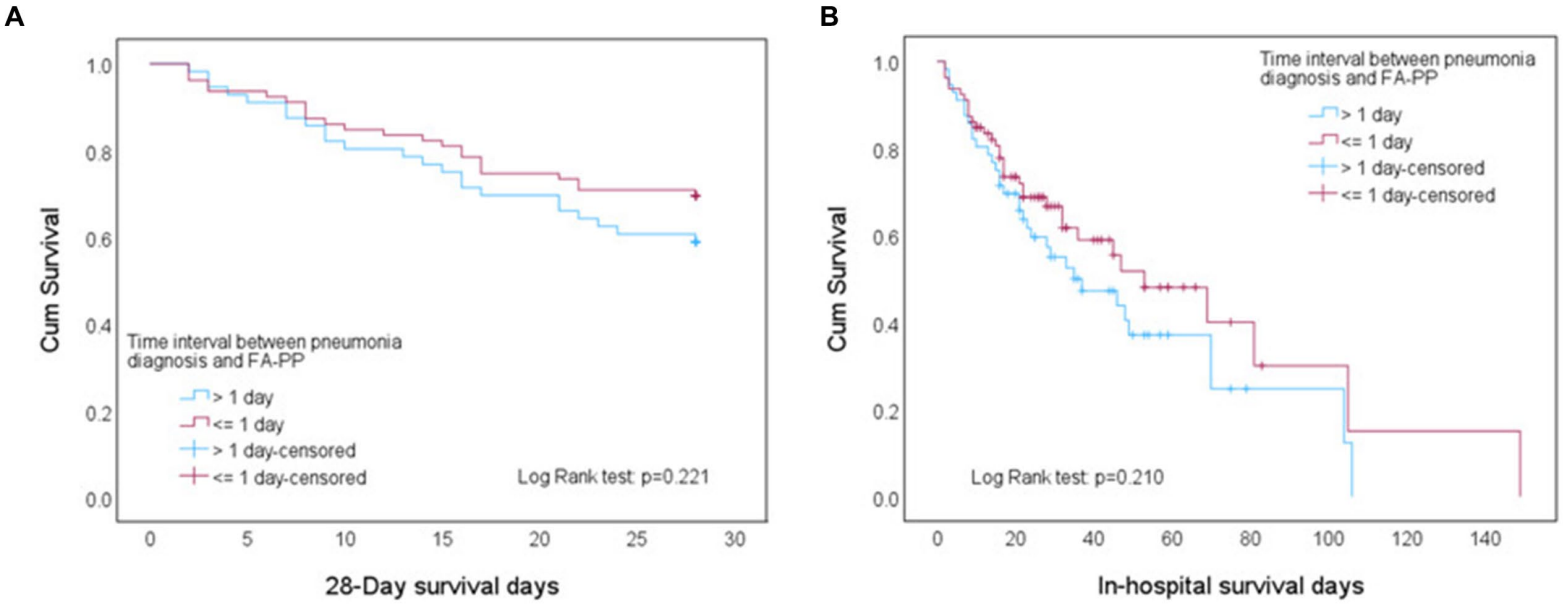
Kaplan–Meier curves showing the **(A)** 28-day survival rate and **(B)** in-hospital survival rate.

According to Kaplan–Meier survival analysis, the early test group, who underwent the FA-PP test within 1 day, did not show statistically significant differences in survival at 28 days and during hospitalization (Log Rank test: *p* = 0.221 and 0.210, respectively) (Figure 2). The results indicated that early FA-PP testing did not provide a survival benefit for the patients with severe pneumonia combined with acute respiratory failure.

## 4 Discussion

This retrospective study showed that among pneumonia patients requiring ventilator support, FA-PP testing, regardless of when it was administered during the treatment process, did not significantly improve patient survival rates.

Prior investigations have emphasized the importance of using the FA-PP test as a valuable tool for accurately diagnosing pathogenic bacteria and selecting appropriate antibiotics for patients with pneumonia (8, 9, 11). Monard et al. further evaluated a multiplex PCR test, which offered a promising approach to the early adaptation of antimicrobial therapy in adult patients with pneumonia (8). The capacity of this technology to simultaneously detect multiple pathogens enhances the precision of treatment decisions, aligning with the broader goal of reducing antibiotic misuse. Prior research by on the BioFire^®^ FilmArray^®^ Pneumonia Panel Gastli et al. demonstrated the capacity of this technique for rapid and accurate identification of pneumonia-causing bacteria (9). This tool can significantly improve bacteriological documentation, which is vital for pneumonia treatment. Buchan et al. compared the BioFire^®^ FilmArray^®^ Pneumonia Panel with conventional diagnostic methods (11), showing the potential impact of antimicrobial stewardship on adults with lower respiratory tract infections, highlighting the importance of this technology in optimizing antibiotic use. Collectively, these studies underscore the significance of enhancing pneumonia diagnostics to provide more efficient and precise diagnostic tools. The use of multiplex PCR and the BioFire^®^ FilmArray^®^ Pneumonia Panel holds promise for guiding personalized, rapid, and effective treatment decisions. These advancements address the challenges associated with pneumonia management and contribute to improved patient care, while reducing unnecessary antibiotic use.

Rapid and accurate diagnosis and treatment are crucial for patients with sepsis. However, in our hospitals, sputum bacterial culture results typically take an average of 3 ± 2.6 days, and this delay can allow exacerbation of the underlying disease. In contrast, the FA-PP testing offers a faster alternative, with an average turnaround time of only 1 h. The rapidity of this test is of paramount importance in sepsis management. According to the Surviving Sepsis Campaign 2021 guidelines, for patients with suspected septic shock or a high likelihood of sepsis, antibiotics should be administered immediately, ideally within 1 h and 3 h of recognition, respectively (14). However, traditional bacterial culture methods often require several days to yield specific bacterial infection information, potentially leading to delayed antibiotic treatment. Under such circumstances, the FA-PP testing can provide rapid bacterial infection information, enabling healthcare professionals to make timely decisions regarding the appropriate antibiotic therapy. This not only enhances patient outcomes, but can also facilitate adherence to the recommendations of the Surviving Sepsis Campaign, and strengthen antibiotic stewardship for patients with sepsis. In this retrospective study, we initially hypothesized that for patients with severe pneumonia combined with respiratory failure, early intervention with FA-PP testing during the treatment process would lead to an increase in survival rates due to adjustments in antibiotic therapy.

The judicious use of antibiotics can drastically reduce patient mortality. A previous study found that patients in the ICU with an APACHE-II score of >30 had an average mortality rate of 73% (15). In 2011, Richards et al. reported that patients with community-acquired pneumonia combined with severe sepsis and an APACHE-II score > 25 had an in-hospital mortality rate of 48.2% (16). More recently, a study of 6,374 critically ill patients found that non-survivors had an average APACHE-II score of 19.8 ± 6.1 on the first day after admission (17). Our study revealed that, among patients with severe pneumonia with an average APACHE-II score of 30.62 ± 8.46, the use of FA-PP testing to guide antibiotic therapy resulted in an overall mortality rate of 50.4%. In the multivariate logistic regression analysis (Table 3), FA-PP testing was also found to be non-significantly associated with mortality (*p* = 0.067). Additionally, the typical treatment duration for pneumonia is approximately 14 days. We attempted to determine whether the timing of FA-PP testing during the treatment process affected survival rates at different time points. However, Kaplan–Meier survival analysis revealed that neither early nor late FA-PP testing resulted in significant differences in 28-day survival rates or in-hospital survival rates (Figure 2). This phenomenon may be attributed to the numerous factors affecting pneumonia prognosis, including age, sex, number of organ dysfunctions, and underlying diseases (18, 19). Through multivariate analysis, our retrospective study demonstrated that the APACHE-II score and history of malignancy were significant independent predictors of mortality among patients with severe pneumonia. Additionally, the financial situation of the patient’s family can also influence the direction of medical care.

Although this retrospective study found a negative result for FA-PP testing, showing no benefit in patient survival rates, it does provide a clearer role for FA-PP testing. The FA-PP test can help reduce the use of broad-spectrum antibiotics and unnecessary antibiotic treatments without increasing the risk of treatment failure (8, 20). Additionally, the FA-PP test can lower overall healthcare expenditures and social costs (21). A recent meta-analysis found that, compared to traditional diagnostic methods, the use of the FA-PP test in cases of viral pneumonia resulted in a shorter diagnosis time (mean difference − 24.22 h, 95% CI −28.70 to −19.74 h), leading to improved medication control and a consequent reduction in hospitalization days (mean difference − 0.82 days, 95% CI −1.52 to −0.11 days) (22).

## 5 Limitations

This study had several limitations that warrant consideration. First, it should be noted that the research design is retrospective and confined to a single center, which may curtail the generalizability of the findings to broader populations. Second, the sample size, although adequate for the study’s scope, was relatively small. Third, an important aspect not addressed in this study is the evaluation of the influence of various pathogens on patient outcomes, which may merit exploration in future endeavors. In addition, this study lacked a control group of patients who did not undergo the FA-PP testing, which may provide valuable comparative insights.

## 6 Conclusion

Overall, the results of this study suggest that the FA-PP test appears to have no impact on the mortality rate of patients with severe pneumonia and respiratory failure, regardless of whether it is performed early or later. However, a history of malignancy and a higher APACHE-II score remain important independent risk factors for mortality. Further research is required to validate these findings and explore the impact of different pathogens on patient outcomes.

## Informed consent statement

Patient consent was waived due to the use of anonymized data.

## Data availability statement

The original contributions presented in the study are included in the article/supplementary material, further inquiries can be directed to the corresponding author.

## Ethics statement

The studies involving humans were approved by Research Ethics Review Committee Far Eastern Memorial Hospital. The studies were conducted in accordance with the local legislation and institutional requirements. Written informed consent for participation was not required from the participants or the participants’ legal guardians/next of kin because this retrospective single-center cohort study was conducted at a medical center in Taiwan between July 1, 2021, and July26, 2022. This study used anonymous data and was approved by the Medical Ethics Committee of the Far Eastern Memorial Hospital (approval number: 111211-E).

## Author contributions

J-HZ: Formal analysis, Funding acquisition, Investigation, Methodology, Writing – original draft, Writing – review & editing. S-FC: Data curation, Formal analysis, Methodology, Software, Validation, Writing – review & editing. P-HW: Formal analysis, Investigation, Resources, Supervision, Writing – review & editing. C-JY: Methodology, Supervision, Validation, Data curation, Formal analysis, Writing – review & editing. Y-HL: Conceptualization, Methodology, Software, Validation, Writing – review & editing. M-YC: Project administration, Resources, Supervision, Writing – review & editing. H-TC: Funding acquisition, Supervision, Writing – review & editing.
